# Molecular Sensory Analysis Confirms Wood Smoke Exposure as a Source of Smoky Off-Flavors in Fermented Cocoa

**DOI:** 10.1021/acs.jafc.5c06046

**Published:** 2025-07-25

**Authors:** Franziska Krause, Martin Steinhaus

**Affiliations:** 1 Technical University of Munich, TUM School of Natural Sciences, Department of Chemistry, Technical University of Munich, Lichtenbergstraße 4, Garching 85748, Germany; 2 Leibniz Institute for Food Systems Biology at the Technical University of Munich (Leibniz-LSB@TUM), Lise-Meitner-Straße 34, Freising 85354, Germany

**Keywords:** *Theobroma cacao* L., wood smoke off-flavor, gas chromatography−olfactometry (GC−O), 2,6-dimethoxyphenol, odor activity value (OAV)

## Abstract

Among the off-flavors occasionally found in fermented cocoa, a smoky taint is common. While major contributors to the off-flavor are already known, their source has not been fully clarified: wood smoke contact during drying and overfermentation are currently discussed. Odorant screening by gas chromatography–olfactometry and aroma extract dilution analysis applied to a cocoa sample smoked in a worst-case scenario confirmed 2-methoxyphenol, 3- and 4-methylphenol, 3- and 4-ethylphenol, and 3-propylphenol as important smoky odorants and additionally suggested 2,6-dimethoxyphenol as a potential off-flavor compound. Quantitation and odor activity value calculation of the compounds in fermented cocoa with authentic wood smoke contact in the origin revealed 2-methoxyphenol, 4-methylphenol, and 3-ethylphenol as the most potent smoky odorants. Their distribution between nibs and husks showed considerable diffusion into the nibs; thus, husk removal during further processing cannot guarantee a substantial reduction of the smoky compounds.

## Graphical abstract

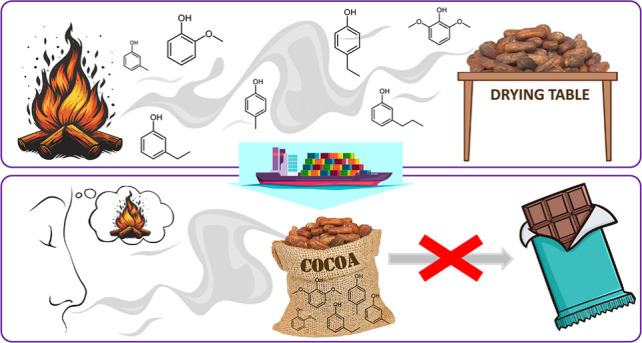

## Introduction

Chocolate products are popular snacks and desserts, eaten worldwide, regardless of age or social class. The basis for chocolate products is the seeds of the tropical cocoa tree, L., known as cocoa beans. The tree’s origins can be traced back to Latin America; however, it has since become distributed worldwide. Today, the main agricultural production region for cocoa is West Africa, but Southeast Asia and Latin America producers also contribute to the world market.^1–3^

The flowers of the cocoa tree grow directly on the trunk and big branches. They develop into mature fruits known as pods. The pods vary considerably in size, color, and shape, and contain the cocoa beans covered by a viscous, sugary, and adhesive cocoa pulp.^4,5^ After harvest, beans and adherent pulp are fermented in heaps or wooden boxes. The fermentation lasts 2 to 10 days, depending on the fermentation method and the cocoa variety. During fermentation, a wide range of microorganisms, including yeasts, lactic acid bacteria, and acetic acid bacteria, convert the sugars of the pulp into ethanol and organic acids, causing the temperature to rise. Diffusion of acetic acid into the beans causes the death of the embryo. Polyphenols and proteins undergo oxidative polymerization, resulting in the typical brown color, reduced bitterness, and reduced astringency.^6–8^ After fermentation, the cocoa beans are spread out and regularly turned to initiate the drying process. At the end of the drying period, the moisture content of the beans should be <8% to avoid microbial growth during storage and shipment. Depending on climatic conditions, sun-drying might not be sufficient, thus making additional artificial drying necessary.^6,9^ Fermented and dried cocoa beans are shipped worldwide.

In the target country, most cocoa beans are processed into chocolate. A typical chocolate manufacturing process starts with the beans being crushed and deshelled to obtain cocoa nibs. During the following roasting step, aroma precursors formed in fermentation react to characteristic chocolate odorants. Roasted cocoa nibs are ground and milled to produce cocoa liquor. Some cocoa liquor is further processed into cocoa butter and cocoa powder. For chocolate production, cocoa liquor is mixed with sugar and further milled. Potential additional ingredients include cocoa butter, emulsifiers, flavorings, and milk powder.

Chocolate is particularly valued for its characteristic aroma and smooth mouthfeel. The molecular background of cocoa and chocolate aroma has been intensively studied, leading to the identification of ∼20–30 key odorants.^10–14^ Some other compounds, however, have the potential to impart atypical or even unpleasant odor notes. Occasionally, batches of fermented cocoa are tainted with smoky, moldy, fecal, cheesy, mushroom-like, or coconut-like off-flavors. The odorants responsible for these flavor deviations have recently been identified.^15–17^ For example, the smoky off-flavor was attributed to phenols such as 2-methoxyphenol, 3- and 4-methylphenol, 3- and 4-ethylphenol, and 3-propylphenol.^17^ However, no attempt was made in this study to clarify the origin of the off-flavor compounds. Nevertheless, wood smoke contact during artificial drying has been discussed as a possible source, as well as overfermentation.^1,2,18–24^ So far, no study has shown a clear relationship between the observed smoky off-flavors and the proposed underlying causes.

To fill this gap, our study first aimed to screen a sample of fermented cocoa beans that had been intentionally exposed to extreme levels of wood smoke in an experimental setting for odor-active compounds by gas chromatography–olfactometry (GC–O)^25^ and aroma extract dilution analysis (AEDA).^26^ This worst-case scenario should ensure that no compounds contributing to the smoky off-flavor were overlooked. Targeted quantitation of the resulting potential off-flavor compounds was then extended to two cocoa samples with authentic wood smoke contact during drying in the origin. The low number of these samples was due to the problem that producers of high-quality cocoa would not allow experiments with wood smoke contact on-site in their processing facilities, while producers who used wood fires in the drying process with insufficient dissipation of the smoke tend not to admit their inappropriate processes. Quantitation was separately applied to nibs and husks, as a recent study has shown that cocoa odorants may be unevenly distributed between both.^16^

## Materials and Methods

### Cocoa

A consortium of the German Chocolate Industry provided the cocoa beans. A sample of fermented cocoa beans, originating from Ecuador, served as an off-flavor-free reference. The experimentally smoked sample was obtained from the reference cocoa by 20 min intense contact with wood smoke. This was achieved by pyrolysis of beechwood pellets, whereby the smoke produced was passed through a vessel containing the reference cocoa beans.

Samples of fermented cocoa with a confirmed history of correct fermentation and authentic wood smoke contact during the drying process originated from Indonesia (sample 1) and Papua New Guinea (sample 2) and were provided by Rausch (Berlin, Germany). After monitoring the cocoa production on-site and sensory assessment of the product, the company eventually rejected the samples due to the smoky off-flavor.

### Chemicals

The following reference odorants were purchased from commercial sources: 3-methylphenol, 4-ethylphenol (Merck; Darmstadt, Germany), 2,6-dimethoxyphenol, 3-propylphenol, 3-ethylphenol (Thermo Fisher Scientific; Waltham, Massachusetts, USA), 2-methoxyphenol (TCI; Tokyo, Japan), and 4-methylphenol (ABCR; Karlsruhe, Germany). The following isotopically substituted odorants were synthesized according to procedures from the literature: (^2^H_3_)-2-methoxyphenol,^27^ (^2^H_2_)-4-ethylphenol,^17^ (^2^H_5–8_)-2,6-dimethoxyphenol,^28^ and (^2^H_11_)-3-propylphenol.^29^ (^2^H_7_)-4-methylphenol was purchased from Merck. Dichloromethane was from CLN (Langenbach, Germany) and, before use, was freshly distilled through a column (120 cm × 5 cm) packed with Raschig rings.

### GC–O/FID

The system used for GC–O analysis consisted of a trace gas chromatograph (Thermo Fisher Scientific; Dreieich, Germany) equipped with a cold on-column injector, a flame ionization detector (FID), a custom-made aluminum sniffing port,^30^ and a DB-FFAP column, 30 m × 0.32 mm i.d., 0.25 μm film thickness (Agilent; Waldbronn, Germany). The carrier gas was helium at a constant flow of 1.0 mL/min. The injection volume was 1 μL. The initial oven temperature of 40 °C was held for 2 min and then increased to 230 °C by 6 °C/min. The final temperature was held for 5 min. A Y-shaped glass splitter connected to the end of the column delivered the effluent through two uncoated but deactivated fused silica capillaries (50 cm × 0.25 mm i.d.) simultaneously to the FID (250 °C base temperature) and the sniffing port (230 °C base temperature). GC–O analyses were performed by trained assessors with >3 months of experience in GC–O of odorant mixtures. Training included weekly sensory testing on odorant recognition, flavor language, and anosmia.^25^ During a GC–O analysis, the FID chromatogram was plotted by a recorder. The assessor placed the nose directly above the sniffing port and marked the position of each odor-active region together with the odor description in the FID chromatogram. The odorants’ retention indices (RIs) were calculated by linear interpolation from the retention times of the odor-active regions and the retention times of adjacent *n*-alkanes.

### Heart-Cut GC–GC–HRMS

The two-dimensional heart-cut GC–GC–high-resolution mass spectrometry (HRMS) system used for structure elucidation and quantitation consisted of two Trace 1310 gas chromatographs (Thermo Fisher Scientific) connected with a Deans switch (Trajan; Ringwood, Australia), and a high-resolution Q Exactive GC Orbitrap mass spectrometer (Thermo Fisher Scientific). The first GC was equipped with a TriPlus RSH autosampler, a programmed temperature vaporizing (PTV) injector, and a DB-FFAP column, 30 m × 0.32 mm i.d., 0.25 μm thickness (Agilent); an FID (250 °C base temperature) and a custom-made sniffing port served as monitor detectors. The carrier gas was helium at a constant flow of 1.0 mL/min. The injection volume was 1–2 μL. The initial oven temperature of 40 °C was held for 2 min and then increased to 230 °C by 6 °C/min. The final temperature was held for 5 min. The end of the column was connected to the Deans switch, which directed the column effluent time-programmed through uncoated but deactivated fused silica capillaries (0.25 mm i.d.) either to the monitor detectors or via a heated hose (250 °C) to a liquid nitrogen-cooled trap. The trap was connected to the column in the second GC, which was a DB-1701 or DB-FFAP column, 30 m × 0.25 mm, i.d., 0.25 μm film thickness (Agilent). The initial temperature of the second oven was 40 °C, held for 2 min, and then increased to 230 °C by 6 °C/min. The final temperature was held for 5 min. The end of the second GC column was connected to the mass spectrometer operated in high-resolution mode. Electron ionization (EI) and chemical ionization (CI) modes were applied for structure assignment using scan ranges of *m*/*z* 35–260 and *m*/*z* 85–260, respectively. The reagent gas used in CI mode was isobutane. Quantitations were performed in the CI mode with a scan range of *m*/*z* 90–280. Data evaluation was accomplished with the Xcalibur software (Thermo Fisher Scientific).

### Aroma Extract Dilution Analysis (AEDA)

Cocoa beans were frozen with liquid nitrogen and preground using a laboratory mill (Retsch; Haan, Germany). The coarse material was ground to a fine powder using a 6875 Freezer Mill (SPEX SamplePrep; Stanmore, UK). A cocoa powder sample (50 g) was stirred with water (80 mL) in an Erlenmeyer flask for 10 min. After the addition of dichloromethane (250 mL), the mixture was stirred at ambient temperature overnight, dried over anhydrous sodium sulfate, and filtered. Nonvolatiles were removed by SAFE at 40 °C.^31^ The distillates fully reproduced the typical aroma of the samples when tested on an olfactory test strip after evaporation of the solvent, particularly the smoky off-flavor of the experimentally smoked sample was clearly perceptible. The volatile isolates were concentrated (1 mL), first using a Vigreux column (50 × 1 cm) and then a microdestillation device.^32^

The volatile isolates of the reference sample and the experimentally smoked sample were diluted stepwise 1:2 with dichloromethane until a dilution of 1:8192. The undiluted volatile isolates as well as each diluted sample were analyzed by GC–O/FID. Each odor-active region in the chromatogram was assigned a flavor dilution (FD) factor corresponding to the dilution factor of the highest diluted sample in which any of two assessors with complementary olfactory abilities^25^ perceived the odor.

Structure assignments of the odorants were based on the odor quality and the RI obtained during GC–O^33^ and comparison of the data with data obtained from authentic reference odorants analyzed in parallel. For smoky odorants, structural assignments were confirmed by parallel GC–GC–HRMS analysis of the cocoa volatile isolates and the corresponding reference odorants.

### Odorant Quantitation

Fermented cocoa beans were first separated into a nibs and a husks fraction using a cocoa breaker and a winnower with a vibratory feeder (Commodity Processing Systems; Colchester, UK). Breaker and winnower were thoroughly cleaned after each sample to prevent carryover. The nibs and the husks fractions were further purified by manual sorting to obtain a 100% nibs sample and a 100% husks sample. The nibs and husks were separately ground to a fine powder. Powder (0.5–10 g) was stirred with water^34^ (4–20 mL) for 10 min. Dichloromethane (40–200 mL) was added together with stable isotopically substituted odorants used as internal standards. The amount of internal standard varied between 0.02 and 2 μg, depending on the expected target compound concentration and the amount of sample used for the workup. The mixture was stirred overnight, dried, filtered, and nonvolatiles were removed by SAFE.^31^ Volatile isolates were concentrated to a final volume of 200 μL and analyzed with the heart-cut GC–GC–HRMS system. Peak areas corresponding to the analytes and the internal standards were obtained from extracted ion chromatograms using characteristic quantifier ions. Odorant concentrations in the cocoa samples were finally calculated from the area counts, the amount of internal standard added, and the amount of the cocoa sample used for the workup with the help of a calibration line equation. Individual calibration line equations were obtained from odorant/standard mixtures with different concentration ratios (1:10, 1:5, 1:2, 1:1, 2:1, 5:1, and 10:1), which had been analyzed under identical conditions, followed by linear regression. Quantitations were carried out in triplicate. Stable isotopically substituted internal standards, quantifier ions, calibration lines, and individual concentration data used for mean calculations are detailed in the Supporting Information file, Tables S2–S10.

## Results and Discussion

### Odorant Screening

GC–O in combination with a comparative AEDA in parallel applied to the volatile isolates obtained from fermented and dried cocoa beans before (off-flavor-free reference sample) and after smoking in an experimental setting (experimentally smoked sample) resulted in 43 odor-active regions in the chromatogram with FD factors ranging from 4 to 4096 (cf. Supporting Information file, Table S1). Four odor-active regions showed smoky odor characteristics and could be associated with a total of six odor-active compounds (Table 1). With an FD factor of 4096, the most potent odorant in the experimentally smoked sample was smoky, sweet, and gammon-like smelling 2-methoxyphenol. A smoky, phenolic, and leather-like smelling chromatogram region with an FD factor of 256 was assigned to 3- and 4-ethylphenol, and a smoky, sweet, and clove-like smelling region with an FD factor of 128 was ascribed to 2,6-dimethoxyphenol. The fourth region was described as horse stable-like, smoky, and phenolic, showed an FD factor of 32, and could be linked to 3- and 4-methylphenol. All the FD factors of the smoky smelling chromatogram regions were substantially higher in the experimentally smoked sample than in the off-flavor-free reference sample.

**Table 1:** Odorants with Smoky Odor Quality Detected in the Volatile Isolates Obtained from Fermented and Dried Cocoa Beans before (Reference Sample) and after Smoking in an Experimental Setting (Experimentally Smoked Sample)

			FD factor^d^	
odorant(s)^a^	odor^b^	RI (FFAP)^c^	reference sample	experimentally smoked sample^e^
2-methoxyphenol	smoky, sweet, gammon	1881	32	4096
3-ethylphenol/4-ethylphenol^f^	smoky, phenolic, leather	2203	<1	256
2,6-dimethoxyphenol	smoky, sweet, clove	2297	1	128
3-methylphenol/4-methylphenol^f^	horse stable, smoky, phenolic	2106	2	32

a Structure assignments were based on the odor quality and the retention index (RI) obtained during GC–O and the mass spectra in EI and CI mode obtained by GC–GC–HRMS analysis and comparison of the data with data obtained from authentic reference odorants analyzed in parallel.

b Odor quality perceived during GC–O at the sniffing port.

c Retention index on the FFAP column; calculated from the retention times of the odorants and the retention times of the adjacent *n*-alkanes by linear interpolation.

d Flavor dilution factor: dilution factor of the highest diluted cocoa volatile isolate sample in which the odorant was perceived during GC–O analysis by any of two assessors.

e 20 min intense wood smoke contact.

f The isomers were not sufficiently separated to allow assignment of individual FD factors.

Previous studies have already reported that the five compounds 2-methoxyphenol, 3- and 4-ethylphenol, and 3- and 4-methylphenol contribute to smoky off-flavors in cocoa.^17^ In the experimentally smoked sample of the current study, which reflected a worst-case smoking procedure rather than a simulation of an authentic wood smoke contact during cocoa drying, 2,6-dimethoxyphenol was detected as an additional phenolic compound potentially contributing to smoky off-flavors. 2,6-Dimethoxyphenol has been suggested early as a marker of wood smoke contact in cocoa;^22^ however, whether the compound contributes to smoky off-flavors in cocoa has not been clarified.^20,22^

### Concentrations and OAVs of Smoky Odorants

To substantiate the differences between the reference sample and the experimentally smoked sample, all compounds potentially contributing to the smoky off-flavor were quantitated using GC–MS in combination with deuterated odorants as internal standards (cf. Supporting Information file, Table S2). The quantitations included 2-methoxyphenol, 3- and 4-ethylphenol, 2,6-dimethoxyphenol, and 3- and 4-methylphenol, i.e., the six odorants that resulted from the previous screening experiments, and in addition 3- and 4-propylphenol, which had recently been reported in cocoa with smoky off-flavors.^17^ The quantitations were extended to two cocoa samples that had been authentically exposed to wood smoke during the drying process in the origin and exhibited a pronounced smoky off-flavor. Moreover, quantitation was separately applied to nibs and husks for three reasons: (1) An uneven distribution between nibs and husks of the cocoa bean has been demonstrated for different substances including cadmium^35^ and the off-flavor compounds (−)-geosmin and 3-methyl-1*H*-indole.^16^ (2) Given the superficial contact of the cocoa beans with the wood smoke during drying, higher concentrations of smoky off-flavor compounds in the husks than in the nibs could be expected. (3) Subsequent cocoa processing includes a winnowing step to remove husks, while the nibs fraction is further processed. However, a technically unavoidable proportion of husks remains in the nibs fraction. It might still be sufficient to substantially contribute to the total amount of the smoky off-flavor compounds.

The results of the quantitations are detailed in Table 2; individual concentration data used for mean calculations and standard deviations are available in the Supporting Information file, Tables S3–S10. The experimentally smoked cocoa showed substantially higher concentrations than the reference cocoa in the nibs and the husks. The increase in concentration during smoking strongly depended on the individual substance and varied between ∼3-fold and ∼50-fold in the nibs and between ∼9-fold and ∼400-fold in the husks. In agreement with the superficial wood smoke contact, the odorant concentrations in the husks of the smoked sample were higher than those in the nibs—by a factor of 6 to 25.

**Table 2:** Concentrations (μg/kg) of the Odorants with Smoky Odor Quality in Cocoa Nibs and Husks: Reference without Off-Flavor vs. the Experimentally Smoked Cocoa and Two Samples with Authentic Wood Smoke Contact during the Drying Process in the Origin

		reference sample^b^		experimentally smoked sample^b^ ^,^ ^c^		authentic smoke contact sample 1^b^		authentic smoke contact sample 2^b^	
odorant	OTC^a^	nibs	husks	nibs	husks	nibs	husks	nibs	husks
2-methoxyphenol	1.8	37.1	36.0	212	3350	115	159	437	324
4-methylphenol	3.3	5.97	10.7	16.7	242	54.4	75.6	131	97.0
3-methylphenol	19	3.56	11.6	36.0	351	68.8	121	288	216
4-ethylphenol	23	2.78	4.08	11.3	216	58.4	97.3	283	182
3-ethylphenol	2.2	0.411	2.77	7.47	115	12.7	27.7	96.6	50.3
3-/4-propylphenol	2.0^d^	0.823	2.87	4.66	25.5	2.31	7.44	7.90	14.3
2,6-dimethoxyphenol	83	2.28	6.48	106	2630	98.7	446	263	382

a Odor threshold concentration in deodorized cocoa butter;^17^ the OTC of 2,6-dimethoxyphenol was determined in the current study using the method detailed in ref 8.

b Mean values; individual values and standard deviations are available in the Supporting Information file, Tables S3–S10.

c 20 min intense wood smoke contact.

d OTC of 3-propylphenol; the OTC of 4-propylphenol amounts to 3700 μg/kg.^17^

The comparison of the odorant concentrations between the experimentally smoked sample and the two samples with authentic wood smoke contact revealed two interesting facts: (1) some of the odorant concentrations in the nibs were in the same range (e.g., 2-methoxyphenol and 2,6-dimethoxyphenol), some were higher in the authentic samples (e.g., 4-ethylphenol was 25 times higher in authentic smoke contact sample 2 than in the experimentally smoked sample), and (2) the concentration differences between nibs and husks in the two samples with authentic wood smoke contact was way less pronounced (factors of 0.5 to 4.5) than in the experimentally smoked sample (6 to 25). Authentic smoke contact sample 2 showed even higher concentrations of the majority of compounds, namely, 2-methoxyphenol, 3- and 4-ethylphenol, and 3- and 4-methylphenol, in the nibs than in the husks.

A potential explanation for the different odorant distributions between nibs and husks in the experimentally smoked sample compared to the authentic smoke contact samples is the different moisture content of the beans during the wood smoke contact. The cocoa beans subjected to the experimental smoking process were already fully dried, which may have substantially hindered diffusion of the odorants through the husks into the nibs. In contrast, the authentic smoke contact samples faced wood smoke contact when considerably moist. These samples were processed during the rainy season in the origin. Cloudy skies and high air humidity prevented natural drying; thus, artificial heat generated by wood fires was applied. If, in such a setting, the wood smoke is not sufficiently dissipated, then it can contaminate the cocoa beans when they are still considerably moist. This may have enabled the off-flavor compounds to diffuse into the nibs over the whole drying period of several days. However, this explanation is speculative and further experiments are required in the future to fully clarify the impact of the moisture content in the cocoa beans at the time of wood smoke contact on off-flavor compound concentration and distribution. These experiments may additionally address other parameters such as the type of wood and the intensity and duration of the wood smoke contact.

When discussing off-flavor compound concentrations, it is of utmost importance to put them into relation to the respective odor threshold concentrations (OTCs). As shown in Table 2, column 2, the smoky off-flavor compounds substantially differ in their OTCs, which cover a range between 1.8 μg/kg for 2-methoxyphenol and 83 μg/kg for 2,6-dimethoxyphenol. A parameter allowing for the simultaneous evaluation of concentration differences (within a compound) *and* the potential to impact the overall aroma (not only within a compound but even, though with limitations, between compounds) is the odor activity value (OAV). The OAV is calculated from the odorant concentration in the sample divided by the OTC.

The OAV data corresponding to the concentration data in Table 2 are available in Table 3. The overall lowest OAVs were found for the reference cocoa sample without off-flavor. In the nibs of this sample, five of the seven compounds showed OAVs <1, i.e., their concentrations were below the OTCs. Nevertheless, in agreement with previous studies,^11,14,17^ 2-methoxyphenol (OAV 21) was present in amounts clearly above the OTC, and 4-methylphenol (OAV 1.8) was slightly above the OTC. However, these amounts were below the maximum tolerable concentrations suggested previously^17^ and still insufficient to provoke an off-flavor, as indicated by the overall aroma of the reference cocoa sample. Genuine cocoa odorants may have suppressed the off-notes.

**Table 3:** Odor Activity Values (OAVs) of the Odorants with Smoky Odor Quality in Cocoa Nibs and Husks: Reference without Off-Flavor vs. the Experimentally Smoked Cocoa and Two Samples with Authentic Wood Smoke Contact during the Drying Process in the Origin

	reference sample		experimentally smoked sample		authentic smoke contact sample 1		authentic smoke contact sample 2	
odorant	nibs	husks	nibs	husks	nibs	husks	nibs	husks
2-methoxyphenol	21	20	120	1900	64	89	240	180
4-methylphenol	1.8	3.2	5.1	73	16	23	40	29
3-methylphenol	<1	<1	1.9	18	3.6	6.4	15	11
4-ethylphenol	<1	<1	<1	9.4	2.5	4.2	12	7.9
3-ethylphenol	<1	1.3	3.4	52	5.8	13	44	23
3-/4-propylphenol^a^	<1	1.4	2.3	13	1.2	3.7	3.9	7.2
2,6-dimethoxyphenol	<1	<1	1.3	32	1.2	5.4	3.2	4.6

a OAVs were calculated with the OTC of 3-propylphenol (cf. Table 2).

Another factor that influences the impact of an off-flavor compound is its exact odor quality. At the same OAV level, a compound with a very unpleasant odor note may be more offensive than a compound with a less unpleasant character. This may also influence the perception of the smoky cocoa odorants in the current study. Although a smoky odor note characterizes all compounds in Tables 1–3, they differ in their nuances. For example, 2-methoxyphenol with its somewhat sweet kind of smokiness may be less offensive and thus contribute less to the off-flavor than, e.g., 4-methylphenol with its unpleasant horse dung-like note (cf. Table 1). This may put into perspective that in all nibs and husks samples, 2-methoxyphenol showed the highest OAVs among all compounds investigated. However, in the off-flavor samples, additionally, 4-methylphenol, 3-ethylphenol, and 2,6-dimethoxyphenol showed OAVs >1, and the 2-methoxyphenol concentration was consistently beyond the previously suggested maximum tolerable concentration.^17^ In agreement with the previous study,^17^ 4-methylphenol and 3-ethylphenol always showed the second and third highest OAVs after 2-methoxyphenol, suggesting their substantial role in the overall off-flavor. By contrast, the propylphenols are likely of minor importance for the off-flavor. The concentration of 3- and 4-propylphenol was determined as a sum, and the corresponding OAVs provided in Table 3 were—in the sense of a worst-case concept (i.e., assuming 100% 3-propylphenol)—in the first instance calculated with the lower OTC of 3-propylphenol (2.0 vs 3700 μg/kg). Considering, however, that a substantial percentage of the sum is attributable to the virtually odorless 4-propylisomer,^17^ it becomes clear that even the sum of the isomers (25.5 μg/kg max) is far below its OTC of 3700 μg/kg. Conservatively interpreted, 3-propylphenol, if at all, could contribute only marginally to the off-flavor.

To better visualize the effect of husk removal on the amount of the off-flavor compounds, their absolute distribution between nibs and husks was calculated from the relative amounts of nibs and husks in the cocoa beans (80:20; m/m) and the respective concentrations (Supporting Information, Table S11). The results for the major off-flavor compounds 2-methoxyphenol, 4-methylphenol, 3-methylphenol, 4-ethylphenol, and 3-ethylphenol are depicted in Figure 1. Unlike the experimentally smoked sample, in both samples with authentic wood smoke contact, the major part of the off-flavor compounds was localized in the nibs. Thus, even if performed very effectively, winnowing cannot substantially reduce the smoky off-flavor compounds.

**Figure 1:**
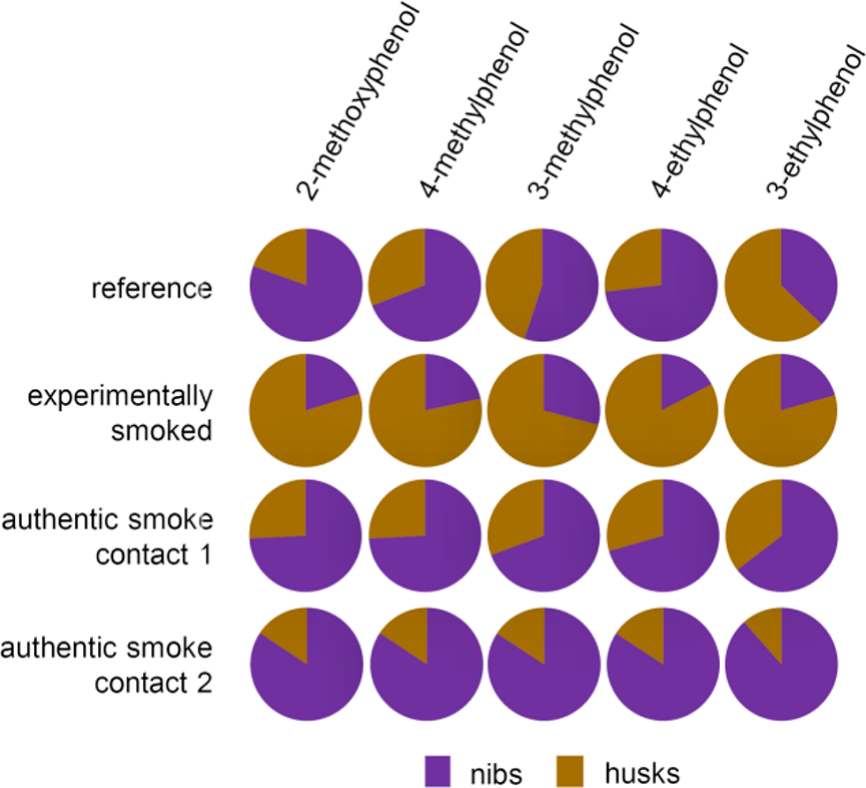
Odorant distribution (m/m) between the nibs and the husks of the cocoa beans: reference cocoa without off-flavor vs experimentally smoked cocoa and cocoa with authentic wood smoke contact 1 and 2. Data were calculated from the mean concentrations (*n* = 3) in nibs and husks (cf. Table 2) and a gravimetric nibs/husks ratio of 80/20 (cf. Supporting Information file, Table S11).

In conclusion, this study confirmed wood smoke exposure as one source of smoky off-flavors in fermented cocoa and clarified the underlying compounds. A comparative AEDA applied to a fermented cocoa sample that had been intentionally exposed to extreme levels of wood smoke in an experimental setting confirmed previously identified off-flavor compounds 2-methoxyphenol, 4-methylphenol, 3-ethylphenol, 3-methylphenol, 4-ethylphenol, and 3-propylphenol^17^ and revealed 2,6-dimethoxyphenol as an additional smoky smelling compound. Quantitative analyses, which additionally included two cocoa samples with a confirmed history of correct fermentation and authentic wood smoke contact during drying in the origin, followed by OAV calculations, suggested that particularly 2-methoxyphenol, 4-methylphenol, and 3-ethylphenol contributed to the smoky off-flavor. 2,6-Dimethoxyphenol showed the overall highest difference between the smoky samples and the reference without off-flavor (>100 times between the reference nibs and the nibs of authentic smoke contact sample 2) and has not yet been reported from overfermented cocoa.^1,21^ 2,6-Dimethoxyphenol may thus be suitable as a marker compound for wood smoke contact, as previously suggested by Lehrian et al.,^23^ but due to its relatively high OTC is unlikely to have a substantial impact on the off-flavor. In the samples with authentic wood smoke contact, the major part of the off-flavor compounds had already diffused into the nibs. Consequently, husk removal by winnowing cannot substantially reduce the smoky off-flavor compounds.

## Supplementary Material



## Notes

Odorants detected in the volatile isolates obtained from fermented and dried cocoa beans before and after smoking in an experimental setting (complete data); stable isotopically substituted internal standards, quantifier ions, and calibration lines used in the quantitation assays; individual concentration data used for mean calculations and standard deviations; and distribution of smoky odorants in nibs and husks (PDF)

## Abbreviations

AEDA: aroma extract dilution analysis
CI: chemical ionization
EI: electron ionization
FD: flavor dilution
FID: flame ionization detector
GC: gas chromatography
GC–GC–HRMS: gas chromatography–gas chromatography–high-resolution mass spectrometry
GC–O: gas chromatography–olfactometry
GC–O/FID: gas chromatography–olfactometry/flame ionization detector
OAV: odor activity value
OTC: odor threshold concentration
PTV: programmed temperature vaporizing
RI: retention index
SAFE: solvent-assisted flavor evaporation
