# Supplementary material for: Molecular Sensory Analysis Confirms Wood Smoke Exposure as a Source of Smoky Off-Flavors in Fermented Cocoa
Source: J Agric Food Chem. 2025 Jul 25;73(31):19663–9. doi: 10.1021/acs.jafc.5c06046 (PMC12333360; doi:10.1021/acs.jafc.5c06046)
Supplement: Supplementary file 1 [file jf5c06046_si_001.pdf]

Supporting Information

Molecular Sensory Analysis Confirms Wood Smoke Exposure as a Source of Smoky Off-Flavors in Fermented Cocoa

Franziska Krause[1,2] and Martin Steinhaus[2,1,*]

[1] Technical University of Munich, TUM School of Natural Sciences, Department of Chemistry, Lichtenbergstraße 4, 85748 Garching, Germany

[2] Leibniz Institute for Food Systems Biology at the Technical University of Munich (Leibniz-LSB@TUM), Lise-Meitner-Straße 34, 85354 Freising, Germany

*Email: m.steinhaus.leibniz-lsb@tum.de

Overview

Table S1. **Odorants Detected in the Volatile Isolates Obtained from Fermented and Dried Cocoa Beans before (Reference Sample) and after Smoking in an Experimental Setting (Experimentally Smoked Sample)**

Table S2. **Stable Isotopically Substituted Internal Standards, Quantifier Ions, and Calibration Lines Used in the GC–GC–HRMS Quantitations**

Table S3. **Concentration of Smoky Off-Flavor Compounds in the Nibs of the Reference Cocoa without Off-Flavor (Reference Sample)**

Table S4. **Concentration of Smoky Off-Flavor Compounds in the Husks of the Reference Cocoa without Off-Flavor (Reference Sample)**

Table S5. **Concentration of Smoky Off-Flavor Compounds in the Nibs of the Cocoa Experimentally Exposed to Wood Smoke (Experimentally Smoked Sample)**

Table S6. **Concentration of Smoky Off-Flavor Compounds in the Husks of the Cocoa Experimentally Exposed to Wood Smoke (Experimentally Smoked Sample)**

Table S7. **Concentration of Smoky Off-Flavor Compounds in the Nibs of the Cocoa with Authentic Wood Smoke Contact, Sample 1**

Table S8. **Concentration of Smoky Off-Flavor Compounds in the Husks of the Cocoa with Authentic Wood Smoke Contact, Sample 1**

Table S9. **Concentration of Smoky Off-Flavor Compounds in the Nibs of the Cocoa with Authentic Wood Smoke Contact, Sample 2**

Table S10. **Concentration of Smoky Off-Flavor Compounds in the Husks of the Cocoa with Authentic Wood Smoke Contact, Sample 2**

Table S11. **Distribution of Odorants with Smoky Odor Quality between Cocoa Nibs and Husks: Reference without Off-Flavor vs. the Experimentally Smoked Cocoa and Two Samples with Authentic Wood Smoke Contact during the Drying Process in the Origin**

Table S1. Odorants Detected in the Volatile Isolates Obtained from Fermented and Dried Cocoa Beans before (Reference Sample) and after Smoking in an Experimental Setting (Experimentally Smoked Sample)

no.	odorant(s)[a]	odor[b]	RI (FFAP)[c]	FD factor[d] reference sample	FD factor[d] experimentally smoked sample[e]
1	ethyl 3-methylbutanoate	fruity	1072	16	16
2	3-methylbutyl acetate	fruity, banana	1128	32	32
3	unknown	fruity	1183	16	16
4	unknown	fruity	1198	512	512
5	2-/3-methylbutan-1-ol	malty	1209	8	8
6	unknown	musty	1238	1	4
7	unknown	fruity	1260	8	8
8	unknown	fruity	1273	512	512
9	unknown	buttery	1296	16	16
10	1-octen-3-one	mushroom	1307	32	32
11	unknown	earthy, green	1327	32	32
12	2-acetyl-1-pyrroline	popcorn	1340	32	512
13	dimethyltrisulfide	sulfury, cabbage	1384	32	32
14	unknown	green	1414	512	512
15	3-isopropyl-2-methoxypyrazine	earthy	1439	1024	1024
16	acetic acid	vinegar, pungent	1451	32	8
17	3-(methylsulfanyl)propanal	cooked potato	1473	256	512
18	2,3-diethyl-5-methylpyrazine	earthy	1507	8	8
19	3-isobutyl-2-methoxypyrazine	bell pepper	1531	2048	2048
20	unknown	floral	1540	32	32
21	unknown	floral	1552	8	8
22	2-methylpropanoic acid	cheesy	1567	32	32
23	butanoic acid	cheesy	1629	2	8
24	phenylacetaldehyde	floral	1659	128	512
25	2-/3-methylbutanoic acid	sweaty	1673	64	64
26	sotolon	fenugreek, lovage	1676	2048	2048
27	unknown	green	1715	1024	1024
28	unknown	floral	1777	8	8
29	unknown	floral	1800	32	32
30	2-phenylethyl acetate	honey, floral	1832	2048	2048
31	geraniol	rose, citrusy	1862	4	4
32	2-methoxyphenol[f]	smoky, sweet, gammon	1881	32	4096
33	2-phenylethan-1-ol	floral	1931	256	256
35	unknown	floral, hay	1986	8	8
36	*p*-anisaldehyde	woodruff, aniseed	2026	32	512
37	unknown	green, sweet	2054	8	4
38	3-/4-methylphenol[f,g]	horse stable, smoky, phenolic	2106	2	32
39	unknown	musty	2106	128	128
40	3-/4-ethylphenol[f,g]	smoky, phenolic, leather	2203	<1	256
41	unknown	floral, sweet	2268	64	128
42	2,6-dimethoxyphenol[f]	smoky, sweet, clove	2297	1	128
43	phenylacetic acid	honey, beeswax	2610	8	8

[a]Structure assignments were based on the odor quality and the retention index (RI) obtained during GC–O and comparison of the data with data obtained from authentic reference odorants analyzed in parallel. [b]Odor quality perceived during GC–O at the sniffing port. [c]Retention index on the FFAP column; calculated from the retention times of the odorants and the retention times of the adjacent *n*-alkanes by linear interpolation. [d]Flavor dilution factor: dilution factor of the highest diluted cocoa volatile isolate sample in which the odorant was perceived during GC–O analysis by any of two assessors. [e]20 min intense wood smoke contact. [f]In addition to the parameters mentioned in footnote a, structural assignments were confirmed by GC–GC–HRMS analysis of the cocoa extract and corresponding reference odorants. [g]The isomers were not sufficiently separated to allow assignment of individual FD factors.

Table S2. Stable Isotopically Substituted Internal Standards, Quantifier Ions, and Calibration Lines Used in the GC–GC–HRMS Quantitations

odorant(s)	internal standard	quantifier ions (*m*/*z*)		calibration line equation	R^2
		analyte	standard		
2-methoxyphenol	($^2H_3$)-2-methoxyphenol	125.0597	128.0785	y = 1.0704x − 0.0821[a]	0.999
4-methylphenol	($^2H_7$)-4-methylphenol	109.0638	113.0899<br>114.0962<br>115.1025<br>116.1087<br>117.1150	y = 1.1236x − 0.0979[a]	0.999
3-methylphenol	($^2H_7$)-4-methylphenol	109.0638	113.0899<br>114.0962<br>115.1025<br>116.1087<br>117.1150	y = 1.1586x − 0.3972[a]	0.974
4-ethylphenol	($^2H_{2-3}$)-4-ethylphenol	123.0804	125.0930<br>126.0993	y = 0.8343x − 0.0095[b]	0.999
3-ethylphenol	($^2H_{2-3}$)-4-ethylphenol	123.0804	125.0930<br>126.0993	y = 0.8983x − 0.0532[b]	0.999
3-/4-propylphenol	($^2H_{11}$)-3-propylphenol	137.3074	148.1651	y = 1.2833x − 1.6230[b,c]	0.999
2,6-dimethoxyphenol	($^2H_{5-8}$)-2,6-dimethoxyphenol	155.0703	157.0828<br>158.0891<br>159.0954<br>160.1017<br>161.1079<br>162.1142<br>163.1205	y = 1.9959x − 0.1323[a]	0.999

[a]y = peak area counts analyte / peak area counts standard; x = concentration analyte (µg/kg) / concentration standard (µg/kg). [b]y = peak area counts standard / peak area counts analyte; x = concentration standard (µg/kg) / concentration analyte (µg/kg). [c]Linearization was not possible over the whole concentration ratio range; if the peak area counts of the standard were smaller than the peak area counts of the analyte, the calibration line equation y = 0.4783x + 0.0278 with R^2 = 0.981 was used.

Table S3. Concentration of Smoky Off-Flavor Compounds in the Nibs of the Reference Cocoa without Off-Flavor (Reference Sample)

odorant	concentration (µg/kg)			
	experiment 1	experiment 2	experiment 3	mean ± SD (CV)[a]
2-methoxyphenol	38.6	35.5	37.2	37.1 ± 1.3 (3%)
4-methylphenol	6.46	5.79	5.67	5.97 ± 0.35 (7%)
3-methylphenol	3.92	3.44	3.31	3.56 ± 0.27 (6%)
4-ethylphenol	3.24	2.68	2.42	2.78 ± 0.34 (12%)
3-ethylphenol	0.672	0.246	0.316	0.411 ± 0.186 (45%)
3-/4-propylphenol	0.835	0.892	0.742	0.823 ± 0.062 (8%)
2,6-dimethoxyphenol	3.71	1.81	1.33	2.28 ± 1.03 (45%)

[a]SD, standard deviation; CV, coefficient of variation.

Table S4. Concentration of Smoky Off-Flavor Compounds in the Husks of the Reference Cocoa without Off-Flavor (Reference Sample)

odorant	concentration (µg/kg)			
	experiment 1	experiment 2	experiment 3	mean ± SD (CV)[a]
2-methoxyphenol	34.4	36.8	36.6	36.0 ± 1.1 (3%)
4-methylphenol	10.6	10.5	10.9	10.7 ± 0.2 (2%)
3-methylphenol	11.3	11.4	12.1	11.6 ± 0.4 (3%)
4-ethylphenol	5.80	3.35	3.09	4.08 ± 1.22 (30%)
3-ethylphenol	2.24	1.13	4.95	2.77 ± 1.60 (58%)
3-/4-propylphenol	4.25	2.26	2.10	2.87 ± 0.98 (34%)
2,6-dimethoxyphenol	6.38	7.55	5.51	6.48 ± 0.84 (13%)

[a]SD, standard deviation; CV, coefficient of variation.

Table S5. Concentration of Smoky Off-Flavor Compounds in the Nibs of the Cocoa Experimentally Exposed to Wood Smoke (Experimentally Smoked Sample)

odorant	concentration (µg/kg)			
	experiment 1	experiment 2	experiment 3	mean ± SD (CV)[a]
2-methoxyphenol	224	206	208	212 ± 8 (4%)
4-methylphenol	17.1	15.5	17.4	16.7 ± 0.8 (5%)
3-methylphenol	36.3	38.2	33.5	36.0 ± 1.9 (5%)
4-ethylphenol	13.7	9.29	10.9	11.3 ± 1.8 (16%)
3-ethylphenol	9.36	6.13	6.91	7.47 ± 1.38 (18%)
3-/4-propylphenol	5.14	5.88	2.97	4.66 ± 1.24 (27%)
2,6-dimethoxyphenol	117	114	87.6	106 ± 13 (12%)

[a]SD, standard deviation; CV, coefficient of variation.

Table S6. Concentration of Smoky Off-Flavor Compounds in the Husks of the Cocoa Experimentally Exposed to Wood Smoke (Experimentally Smoked Sample)

odorant	concentration (µg/kg)			
	experiment 1	experiment 2	experiment 3	mean ± SD (CV)[a]
2-methoxyphenol	3350	3340	3360	3350 ± 7 (0%)
4-methylphenol	235	239	251	242 ± 7 (3%)
3-methylphenol	344	360	350	351 ± 6 (2%)
4-ethylphenol	227	217	205	216 ± 9 (4%)
3-ethylphenol	121	112	111	115 ± 5 (4%)
3-/4-propylphenol	24.2	25.5	26.9	25.5 ± 1.1 (4%)
2,6-dimethoxyphenol	2680	2620	2580	2630 ± 43 (2%)

[a]SD, standard deviation; CV, coefficient of variation.

Table S7. Concentration of Smoky Off-Flavor Compounds in the Nibs of the Cocoa with Authentic Wood Smoke Contact, Sample 1

odorant	concentration (µg/kg)			
	experiment 1	experiment 2	experiment 3	mean ± SD (CV)[a]
2-methoxyphenol	116	113	116	115 ± 2 (1%)
4-methylphenol	54.9	54.5	53.9	54.4 ± 0.4 (1%)
3-methylphenol	70.7	66.3	69.3	68.8 ± 1.8 (3%)
4-ethylphenol	55.1	59.1	61.0	58.4 ± 2.4 (4%)
3-ethylphenol	12.2	11.5	14.3	12.7 ± 1.2 (9%)
3-/4-propylphenol	2.65	2.15	2.13	2.31 ± 0.24 (10%)
2,6-dimethoxyphenol	102	102	92.2	98.7 ± 4.6 (5%)

[a]SD, standard deviation; CV, coefficient of variation.

Table S8. Concentration of Smoky Off-Flavor Compounds in the Husks of the Cocoa with Authentic Wood Smoke Contact, Sample 1

odorant	concentration (µg/kg)			
	experiment 1	experiment 2	experiment 3	mean ± SD (CV)[a]
2-methoxyphenol	152	165	162	159 ± 6 (3%)
4-methylphenol	76.7	75.2	75.1	75.6 ± 0.7 (1%)
3-methylphenol	117	123	123	121 ± 3 (2%)
4-ethylphenol	87.2	108	96.6	97.3 ± 8.5 (9%)
3-ethylphenol	29.1	29.6	24.6	27.7 ± 2.2 (8%)
3-/4-propylphenol	10.3	6.59	5.43	7.44 ± 2.08 (28%)
2,6-dimethoxyphenol	501	478	360	446 ± 62 (14%)

[a]SD, standard deviation; CV, coefficient of variation.

Table S9. Concentration of Smoky Off-Flavor Compounds in the Nibs of the Cocoa with Authentic Wood Smoke Contact, Sample 2

odorant	concentration (µg/kg)			
	experiment 1	experiment 2	experiment 3	mean ± SD (CV)[a]
2-methoxyphenol	426	424	462	437 ± 19 (4%)
4-methylphenol	130	128	134	131 ± 3 (2%)
3-methylphenol	280	282	303	288 ± 10 (4%)
4-ethylphenol	286	268	294	283 ± 11 (4%)
3-ethylphenol	88.2	96.0	106	96.6 ± 7.1 (7%)
3-/4-propylphenol	7.56	7.06	9.07	7.90 ± 0.86 (11%)
2,6-dimethoxyphenol	264	260	266	263 ± 2 (1%)

[a]SD, standard deviation; CV, coefficient of variation.

Table S10. Concentration of Smoky Off-Flavor Compounds in the Husks of the Cocoa with Authentic Wood Smoke Contact, Sample 2

odorant	concentration (µg/kg)			
	experiment 1	experiment 2	experiment 3	mean ± SD (CV)[a]
2-methoxyphenol	334	317	319	324 ± 7 (2%)
4-methylphenol	94.7	95.5	101	97.0 ± 2.7 (3%)
3-methylphenol	216	213	219	216 ± 2 (1%)
4-ethylphenol	179	179	187	182 ± 4 (2%)
3-ethylphenol	47.7	50.9	52.4	50.3 ± 2.0 (4%)
3-/4-propylphenol	12.1	14.5	16.3	14.3 ± 1.8 (12%)
2,6-dimethoxyphenol	387	378	382	382 ± 4 (1%)

[a]SD, standard deviation; CV, coefficient of variation.

Table S11. Distribution of Odorants with Smoky Odor Quality between Cocoa Nibs and Husks: Reference without Off-Flavor vs. the Experimentally Smoked Cocoa and Two Samples with Authentic Wood Smoke Contact during the Drying Process in the Origin

odorant	distribution of odorants between nibs and husks (% m/m)[a]							
	reference sample		experimentally smoked sample		authentic smoke contact sample 1		authentic smoke contact sample 2	
	nibs	husks	nibs	husks	nibs	husks	nibs	husks
2-methoxyphenol	81	19	20	80	74	26	84	16
4-methylphenol	69	31	22	78	74	26	84	16
3-methylphenol	55	45	29	71	69	31	84	16
4-ethylphenol	73	27	17	83	71	29	86	14
3-ethylphenol	37	63	21	79	65	35	88	12
3-/4-propylphenol	53	47	42	58	55	45	69	31
2,6-dimethoxyphenol	58	42	14	86	47	53	73	27

[a]Calculated from the mean concentrations in nibs and husks (cf. Tables S3-S10) and a gravimetric nibs/husks ratio of 80/20.
